# Nourishing the Infant Gut Microbiome to Support Immune Health: Protocol of SUN (Seeding Through Feeding) Randomized Controlled Trial

**DOI:** 10.2196/56772

**Published:** 2024-09-02

**Authors:** Clare R Wall, Nicole C Roy, Jane A Mullaney, Warren Charles McNabb, Olivier Gasser, Karl Fraser, Eric Altermann, Wayne Young, Janine Cooney, Robyn Lawrence, Yannan Jiang, Barbara C Galland, Xiaoxi Fu, Jacqueline N Tonkie, Nisha Mahawar, Amy Luisa Lovell

**Affiliations:** 1 Department of Nutrition and Dietetics University of Auckland Auckland New Zealand; 2 High-Value Nutrition National Science Challenge Auckland New Zealand; 3 Department of Nutrition University of Otago Dunedin New Zealand; 4 AgResearch Palmerston North New Zealand; 5 Riddet Institute Massey University Palmerston North New Zealand; 6 Malaghan Institute of Medical Research Wellington New Zealand; 7 BlueBarn Life Sciences Ltd. Palmerston North New Zealand; 8 School of Veterinary Science Massey University Palmerston North New Zealand; 9 Fonterra Palmerston North New Zealand; 10 The New Zealand Institute for Plant and Food Research Ltd Hamilton New Zealand; 11 Department of Statistics University of Auckland Auckland New Zealand; 12 Department of Women's and Children's Health University of Otago Dunedin New Zealand; 13 School of Food and Advanced Technology Massey University Palmerston North New Zealand

**Keywords:** infant, complementary feeding, gut microbiota, immune, complementary food, feeding, gut microbiome, microbiome, immune system, kūmara, infancy, immune development, infant health, prevalence, respiratory tract infections, RTI

## Abstract

**Background:**

The introduction of complementary foods during the first year of life influences the diversity of the gut microbiome. How this diversity affects immune development and health is unclear.

**Objective:**

This study evaluates the effect of consuming kūmara or kūmara with added banana powder (resistant starch) compared to a reference control at 4 months post randomization on the prevalence of respiratory tract infections and the development of the gut microbiome.

**Methods:**

This study is a double-blind, randomized controlled trial of mothers and their 6-month-old infants (up to n=300) who have not yet started solids. Infants are randomized into one of 3 groups: control arm (C), standard kūmara intervention (K), and a kūmara intervention with added banana powder product (K+) to be consumed daily for 4 months until the infant is approximately 10 months old. Infants are matched for sex using stratified randomization. Data are collected at baseline (prior to commencing solid food) and at 2 and 4 months after commencing solid food (at around 8 and 10 months of age). Data and samples collected at each timepoint include weight and length, intervention adherence (months 2 and 4), illness and medication history, dietary intake (months 2 and 4), sleep (diary and actigraphy), maternal dietary intake, breast milk, feces (baseline and 4 months), and blood samples (baseline and 4 months).

**Results:**

The trial was approved by the Health and Disability Ethics Committee of the Ministry of Health, New Zealand (reference 20/NTA/9). Recruitment and data collection did not commence until January 2022 due to the COVID-19 pandemic. Data collection and analyses are expected to conclude in January 2024 and early 2025, respectively. Results are to be published in 2024 and 2025.

**Conclusions:**

The results of this study will help us understand how the introduction of a specific prebiotic complementary food affects the microbiota and relative abundances of the microbial species, the modulation of immune development, and infant health. It will contribute to the expanding body of research that aims to deepen our understanding of the connections between nutrition, gut microbiota, and early-life postnatal health.

**Trial Registration:**

Australian New Zealand Clinical Trials Registry ACTRN12620000026921; https://www.anzctr.org.au/Trial/Registration/TrialReview.aspx?id=378654

**International Registered Report Identifier (IRRID):**

DERR1-10.2196/56772

## Introduction

### Background

The early stages of gut microbiome development are marked by unique temporal microbe uptake, colonization, and selection, all of which have variable functional characteristics throughout time. This carefully managed microbial sequence begins at birth and continues until the microbiome acquires an adult-like makeup and function around 3 years of age [1]. These many stages of microbiome development are increasingly becoming recognized as critical periods for immune and metabolic development, which can impact long-term health [2].

Early-life postnatal nutrition has been shown to be the most important modulator of the gut microbiome, with breast milk seeding and modulating the microbiome with beneficial bacteria [3]. The gut microbiota of breastfed infants is dominated by *Bifidobacterium* species, which proliferate due to the high levels of human milk oligosaccharides in breast milk [4]. The introduction of solid foods, which are largely influenced by physiological development, culture, and tradition, has a significant impact on the composition and functional capacity of the gut microbiome [5-7]. If an infant continues to be breastfed during the complementary feeding (CF) phase, this can also influence changes in microbial patterns [3]. An increase in dietary intake of complex polysaccharides and proteins increases the abundance of microbes capable of their degradation, including those taxa from the Bacteroidaceae, Lachnospiraceae, and Ruminococcaceae families [1]. The change in the diversity of species increases the production of the short-chain fatty acids (SCFAs) butyrate and propionate [8]. SCFAs are probable mediators of microbiota-host interactions that affect infant health due to their physiological effects, including influence on the intestinal barrier function, host metabolism, and immune system [1].

There is a growing body of research on early-life postnatal nutrition and the effects on the development of the microbiome and subsequent immunological outcomes [9,10]. The infant's immune system and microbiota coevolve throughout the first few months of life, starting a 2-way interaction that eventually leads to competence and maturity [6]. Breast milk and vertical maternal transmission of microorganisms are factors that influence colonization patterns and the immune system's development [1]. Understanding how the microbiota and immune system interact with the introduction of complementary foods opens possibilities to enable us to provide suitable dietary recommendations in order to maintain an age-appropriate microbiome for short- and long-term health.

Randomized controlled trials conducted in this age group tend to be predominated by infant formula and isolated probiotic interventions [11-14]. Although these studies have been useful to characterize differences in microbiota according to formula or probiotic type, there are a limited number of controlled trials that have evaluated the effects of complementary foods, their effect on the gut microbiota, and their efficacy in immune health and development in infancy [15]. One of the reasons for this lack of research is probably because of the complexities of designing robust trials using weaning foods and the difficulties of measuring the dynamic changes in breast milk and food intake in this age group [16].

Respiratory tract infections (RTIs) are a common cause of morbidity and mortality in children under the age of 2 years [17,18]. Observational studies of infants have demonstrated associations between the gut microbiome and RTI, and in particular, the positive effect of breastfeeding on reducing the number and severity of infections [10]. However, none of these studies have examined the effects of food and nutrient intake during the CF phase on the gut microbiome and the risk of RTI.

The aim of the SUN (seeding through feeding) randomized controlled trial is to evaluate the effect of consuming kūmara (sweet potato) (K) or kūmara with added banana powder containing resistant starch (RS; K+) compared to the C at 4 months post randomization on the prevalence of RTI and the development of the gut microbiome. We will also explore how changes in the gut microbiome influence immune development. Kūmara (sweet potato) is commonly used in CF and is a source of starch and nondigestible carbohydrates. RS is a carbohydrate that resists digestion and acts as a prebiotic food for beneficial gut bacteria.

### Primary Outcomes

Primary outcomes include the following: (1) a between-group difference in general practitioner (GP)–confirmed RTI at 4 months post randomization; and (2) parent self-report of respiratory infections validated by GP records collected via a daily health record.

### Secondary Outcomes

Secondary outcomes include the following: (1) between-group differences in other measures, including infant health, assessed using daily records of illness (reporting frequency, illness type, medication prescribed, use of antibiotics, and antibiotic course length). Differences in infant fecal microbiome relative abundance and diversity; (2) differences in infant fecal concentrations of SCFAs and intermediate metabolites; (3) differences in immune cell development, as determined by high-dimensional flow cytometry, as well as the magnitude of antibody responses to the oral rotavirus vaccine in infants paired with rotavirus-specific secretory immunoglobulin A (IgA) quantification in breast milk (breastfeeding mothers only); and (4) differences in nighttime sleep, daytime sleep, and activity of infants assessed using actigraphy and sleep dairy.

## Methods

### Study Design

The SUN study is a parallel 3-arm, randomized controlled trial conducted in Auckland, New Zealand. A total of 300 infants who have not yet started CF are randomized using computer-generated randomization sequences in a 1:1:1 ratio to receive 1 of 2 daily prebiotic food interventions (double-blind standard kūmara powder or kūmara powder with banana powder containing RS) or be in the control arm (C) of the study.

### Participants and Recruitment

Infants are being recruited from the general population in urban central and greater areas of Auckland, New Zealand, from January 2022 to January 2024. Recruitment strategies include electronic and print advertising targeted toward postnatal and community groups, media releases, and appropriate social media platforms such as Facebook. All families that show interest in the study receive an email explaining the study in detail and are provided with the study participant information sheet and consent form.

### Ethical Considerations

The study was approved by the Health and Disability Ethics Committee of the Ministry of Health, New Zealand (reference 20/NTA/9). Parents or legal guardians are provided with an information sheet explaining the purpose of the trial; details of the research; specific procedures; benefits and risks; the voluntary nature of enrolment and the ability to opt out of the research at any time. A written informed consent form from the infant’s parents or legal guardian is an inclusion criterion, without which the infant cannot be included in the study.

Participants’ data are stored in a password-protected secure study database and are anonymized for analyses. The University of Auckland standards for data handling will be adhered to for data storage and management. No monetary compensation is provided for being in the trial, but on completion, parents are provided with a gift voucher as a thank you for taking part.

### Inclusion and Exclusion Criteria

Infants are eligible for participation in the study if they are healthy; are between 3 and 6 months of age; were at least 32 weeks gestation at birth; will be introduced to their first complimentary foods at around 6 months of age, and not before 4 months of age, as per the New Zealand Ministry of Health Food and Nutrition Guidelines for Infants [19]; and have parents or legal guardians that can provide written informed consent to participate in the study.

Infants are not eligible for participation if they were born <32 weeks gestation; were small for gestational age (as they may have special dietary requirements); have a developmental disability (ie, autism and intellectual disability); have an illness likely to influence their nutritional status (eg, a chronic illness known to cause malabsorption and any digestive or metabolic disorders); have health conditions that affect feeding; are undergoing treatment with antibiotics; have had any complementary foods at recruitment; are receiving a supplement with a prebiotic or probiotic; are not immunized according to the New Zealand Immunization Schedule; and have parents or legal guardians who have written or spoken English comprehension that is likely to make participation difficult for them.

Infants already taking a probiotic or prebiotic as a supplement are still eligible to participate if parents are willing to stop giving these to their infants.

### Randomization

**Figure 1:**
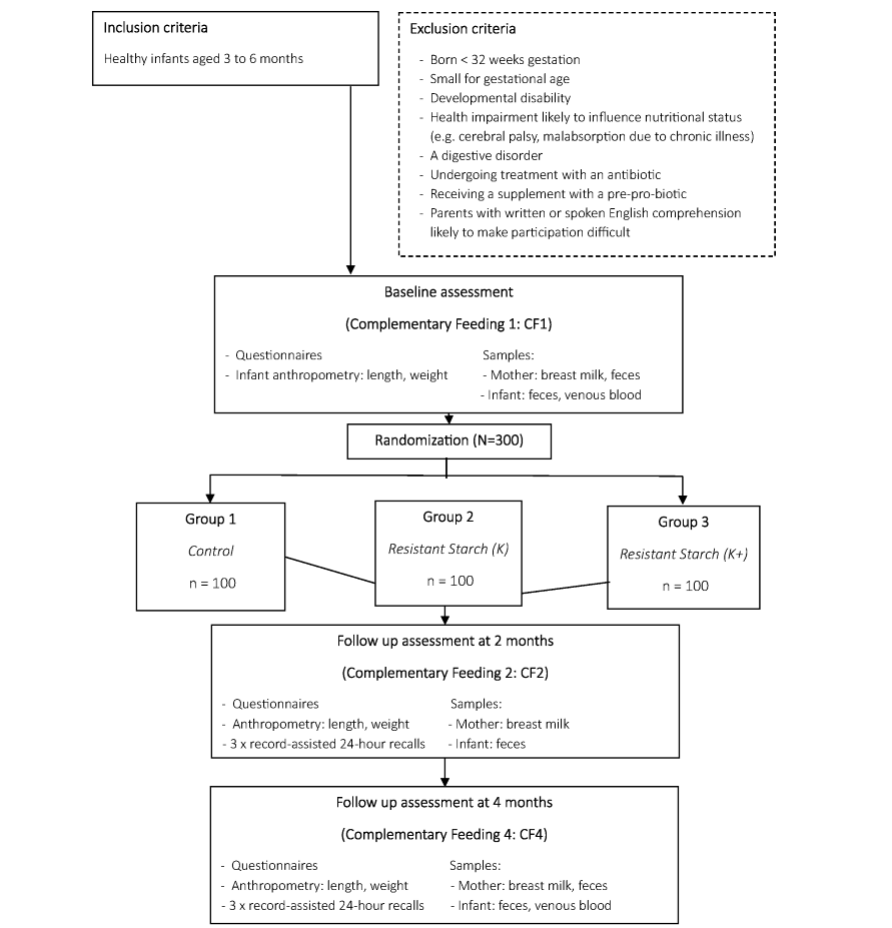
Trial outline.

The trial outline is shown in Figure 1. A “participant randomization form” was designed for the study on REDCap (Research Electronic Data Capture) tools hosted at the University of Auckland, which allows the independent administrator to randomly allocate the participant into the K, K+, or C group. Randomization is stratified by gender (female or male) using block randomization with variable block sizes of 3 or 6. The randomization list was prepared by the trial statistician (YJ) and is maintained by an independent database administrator. The lists are concealed until the point of randomization, and the 2 groups with kūmara intervention will remain double-blinded throughout the trial.

### Intervention

All participants randomized to either the K or K+ intervention groups are supplied with the kūmara powder at no cost to the participant. The kūmara powder is intended to be introduced alongside the first complementary food offered to participating infants and is to be consumed daily for a period of 4 months until the child is approximately 10 months of age. The kūmara powder is packaged in identical 5 g freeze-dried powder sachets and provided as a 2-month supply of study product at each of their clinic visits (baseline and midway through the study). A random number is assigned to each participant kit, using computer-generated sequences. For those randomized to receive one of the kūmara powders (K or K+), both study participants and the researchers will remain blinded throughout the trial.

Any kūmara powder not consumed within the 2-month interval is returned to the research team and recorded at the participant's next clinic visit. Adherence is measured using a daily consumption record completed by parents prospectively and information from a monthly questionnaire on the average amount of the kūmara powder consumed per day in the previous month. Participants randomized into the C group follow general feeding guidelines as per the New Zealand Ministry of Health [19] and receive gift vouchers at their clinic visits to the value of the kūmara powder as a token of appreciation for their continued participation.

### Compliance

Adherence to the kūmara powder is determined using a prospective daily record. Adherence will be defined as the consumption of 5 g of kūmara powder per day (approximately 1 tsp reconstituted with water) on 80% of the days within the monitored interval (ie, the previous month).

### Sample Size

A sample size of 80 participants per group will have 80% power at the 5% significance level (2-sided) to detect an absolute difference of 20% between the K+ and C groups on the primary outcome. This is equivalent to a 25% relative reduction, assuming a retention rate of 80%. To allow for a 20% loss to follow-up, we aim to recruit 300 participants in total (n=100 per group; Figure 1).

### Data Collection

Data collection takes place at baseline, when the child is 4-6 months of age, prior to commencing CF (CF1), and then around 8 months of age (CF2) and around 10 months of age (CF4) post randomization. Prior to the baseline clinic visit, written informed consent is obtained, and parents are given verbal and written information about participation. Questionnaires collecting information on sociodemographic characteristics and participating infant health are completed digitally using REDCap electronic data capture tools hosted by the University of Auckland [20]. At baseline, participants are assessed according to the schedule presented in Table 1 and either provided with a 2-month supply of kūmara powder or the equivalent value in gift vouchers (if randomized to the C group).

**Table 1:** SUN^a^ trial schedule of enrolment, interventions, and assessments.

Timepoint			Study period											
			Enrollment		Allocation		Post allocation							Closeout
							Baseline		CF2		CF4			
Infant age			<4 mo		4-6 mo		≥4-6 mo		≤8 mo		≤10 mo		>10 mo	
**Enrollment**														
	Eligibility screen	✓												
	Informed consent	✓												
	Allocation			✓										
**Intervention**														
	Intervention A													
	Intervention B													
	Control													
**Assessments**														
	Measurements													
	Weight (kg)					✓		✓		✓				
	Length (cm)					✓		✓		✓				
	Questionnaires													
	Demographics	✓												
	Child health and nutrition	✓				✓		✓		✓				
	Maternal health and household	✓				✓				✓				
	Three×record-assisted 24-hour recalls							✓		✓				
	Daily health record					✓		✓		✓				
	Daily intervention adherence					✓		✓		✓				
	FFQ—maternal					✓		✓		✓				
	Sleep questionnaire—mother					✓		✓		✓				
	Biological sample collection													
	Infant													
	Feces					✓		✓		✓				
	Blood					✓				✓				
	5-Day sleep diary (using actigraphy)^b^					✓		✓		✓				
	Mother													
	Feces					✓		✓		✓				
	Breast milk					✓		✓		✓				
Completion and review of electronic trial documentation							✓		✓		✓			
Unblinding and data analyses													✓	

^a^SUN: seeding through feeding.

^b^An actigraph is a noninvasive method of recording infant sleep patterns through movement. The small sensor is fitted around the lower leg using a neoprene strap.

### Demographic Data

At baseline, information on family size (household structure, number of siblings, and presence of pets), parental education, parental occupation, parental ethnicity, living conditions, smoking habits, and family history of disease (eg, common allergens, including pets and pests, respiratory conditions, atopic disease, and chronic disease) is obtained. Information about the participating child includes birth weight and length, mode of delivery, initiation of breastfeeding, feeding patterns since birth, and health records, including hospital admissions and allergy history.

### Illness

Participating infants’ recent health history is captured prospectively via daily records of illness and use of medicines. The incidence and duration of illness or infections, including airway-related illness and gastrointestinal symptoms, are documented. Specific consent is obtained to contact the nominated family GPs to verify records of infection-related visits, medication prescription and use, and hospitalization events throughout the trial [16].

### Measuring Fecal Microbiota, Microbiome, SCFAs, and Rotavirus Vaccine–Specific Antibody Titer

Infant fecal samples are collected at 4-6 months of age (baseline), 8 (CF2), and 10 months of age (CF4) to assess the extent to which the kūmara interventions (K/K+ groups) and CF (all groups) have modulated the infant gut microbiota and to quantify rotavirus vaccine–specific antibody responses. Fecal samples are also analyzed for SCFAs by liquid chromatography-mass spectrometry using modifications to the method reported by Han et al [21]. to investigate the differences in fecal SCFAs and intermediate metabolites in infants in the different feeding interventions. Maternal fecal samples from consenting mothers are collected at baseline, CF2, and CF4 to assess the relationship between maternal and infant gut microbiota composition. The fecal microbial function is assessed using shotgun sequencing of metagenenomic DNA [22] using the Illumina HiSeq platform in conjunction with microbial profiling by pyrotag sequencing of 16S sRNA gene amplicons. Sequencing data will be analyzed using bioinformatics tools such as quantitative insights into microbial ecology [23], Rapid Annotation using Subsystems Technology for metagenomes [24], and Integrated Microbial Genomes [25]. The infant fecal metabolome is measured using high-resolution liquid chromatography–mass spectrometry using the method reported by Abshirini et al [26] to investigate differences in fecal metabolite profiles from the different feeding interventions over time.

### Measuring Rotavirus Vaccine–Specific Antibody Response and Immune Development in Blood

Venipuncture blood samples (this is an optional activity, so only infants whose caregivers will give specific consent) are collected by a trained pediatric phlebotomist at baseline (4-6 months of age) and CF4 (10 months of age). The total collected blood volume is approximately 5 mL (~0.7 mL/kg per 24 hours). Available blood samples will be used to assess the protective antibody response in plasma following oral rotavirus vaccination (administered at 6 weeks and 3 months of age), as well as the frequency of innate and adaptive immune cell subsets by spectral flow cytometry [27].

### Sleep

Quantitative infant sleep data are recorded using a standard ActiGraph (GT3X; Pensacola) worn at each timepoint continuously over 5 days. The actigraph is a watch-sized device fitted around the infant’s lower limb using a neoprene strap and is commonly used to measure sleep via sensing movement (acceleration), where the absence of movement infers sleep and movement infers wake [28,29]. The data will be processed for standard actigraphy sleep variables, inclusive of naps, using the count-scaled algorithm developed in MATLAB (MathWorks) [30]. Parents also record infant sleep in a sleep diary, which captures sleep-wake timing data across a 24-hour period for 5 consecutive days, and the Brief Infant Sleep Questionnaire, which is used to assess sleep patterns, parent perception, and sleep-related behaviors in young children (0-36 months) [31]. Validated parent-completed questionnaires on sleep-related impairment and sleep disturbance are used as a symptom assessment tool. These questionnaires measure 8 items on 5-point scales to rate the frequency of problems related to insufficient sleep and sleep disturbance in the past 7 days [32,33].

### Dietary Intake and Feeding Behaviors

Infant dietary intake is assessed using a 3-day food record at CF2 (infant approximately 8 months of age) and CF4 (infant approximately 10 months of age). Parents are asked to record the participating infant’s food and drink intake over 3 consecutive days, with even distribution across weekdays and weekends at a group level. Parents are instructed on how to complete the 3-day food records and use household measures to describe their infant’s dietary intake. Diet records will be analyzed with FoodWorks (version 10; Xyris Software) using the New Zealand Food Composition database FOODfiles 2018 (version 01).

At baseline (CF1), infant feeding practices such as method (breastfeeding, formula, or mixed feeding) and frequency or amount are recorded. A questionnaire at baseline and diet records at CF2 and CF4 captures the volume of milk intake (expressed breast milk or formula). Breast milk remains an important component of the diet and has important impacts on both the gut microbiota and immune function. Breast milk volume will be estimated using the previously validated methodology, whereby breastfeeding is recorded as time (minutes) and quantity estimated using a conversion factor of 10 mL/min, with a maximum of 10 minutes of breastfeeding or 100 mL of breast milk [34,35].

Habitual maternal food and nutrient intake will be assessed using a semiquantitative food frequency questionnaire, which has previously been validated in the New Zealand setting [36].

### Anthropometry

Anthropometric measurements are taken at baseline when the participating child is around 4-6 months of age (CF1), around 8 months of age (CF2), and around 10 months of age (CF4), using standardized protocols. Measurements include weight, length, and weight for length Z-scores [37].

### Measuring Breast Milk for Rotavirus Vaccine–Specific Antibody Titer and Secretory IgA

Breast milk samples are collected (where possible) from breastfeeding mothers at baseline, CF2, and CF4. Samples will be analyzed for rotavirus vaccine–specific titer quantification and the role of secretory IgA factors in shaping the gut microbiota during infancy and to examine the change in breast milk metabolome with prolonged breastfeeding (ie, beyond 6 months of age) and its continued modulating effect on infant gut microbiota diversity during CF. Sample collection is completed according to a predefined protocol, and breastfeeding mothers will have access to instructional videos on standardized sample collection (by hand expressing or pumping). To account for diurnal variation [38] and compositional differences between fore- and hindmilk [39], breast milk mothers are asked to express by emptying the full breast from the right side between 7 AM and 10 AM and at least 2-3 hours after a previous breastfeed. Wiping the breast with antiseptic (benzalkonium chloride) wipes to limit skin microbial contamination. After mixing the full sample by swirling or gently inverting, 15 mL of breast milk is decanted into a 15-mL falcon tube. Samples are kept in the fridge at home for up to 24 hours before the next clinic appointment. Samples are stored at –80 °C until analysis.

### Statistical Analysis

Participants’ data are stored in a secure study database and imported to SAS software (version 9.4; SAS Institute Inc) for data analysis at the end of the trial. The demographic and baseline characteristics of all randomized participants will be summarized for each group using descriptive statistics. Categorical variables will be described as frequencies and percentages. Continuous variables will be described as mean and SD or median and IQR as appropriate. Descriptive data on important subgroups (gender and ethnicity) will also be presented if a sufficient number of participants are recruited. No formal statistical tests will be conducted to compare the 3 groups at baseline, as recommended by the CONSORT guidelines [40].

There are 3 randomized groups (K+, K, and C) in the trial; however, the primary comparison will be based on the statistical test between K+ and C groups only. Additional comparisons between the other randomized groups will be conducted in secondary analysis using similar statistical models. This is to control the overall type 1 error rate, with the assumption that the proposed sample size will have sufficient power to detect the difference in primary outcome between the K+ and C groups. Primary outcome analysis will be conducted by intention-to-treat (where participants are analyzed according to their allocated treatment arm). The impact of missing data on the primary outcome will be explored in sensitivity analysis using different imputation strategies to test the robustness of the main results.

A secondary analysis of the primary outcomes will be conducted using a per-protocol population, including those participants in the intention-to-treat population who have no major protocol deviations. Inclusion in the per-protocol population and analysis will be based on the degree of intervention compliance, defined as the total number of days where less than the predefined amount of intervention food was consumed per day within the last study month. This information will be determined using a monthly intervention adherence record completed by parents or caregivers. Adherence will be defined as the consumption of 5 g of the intervention food per day on 80% of the days within the monitored interval.

Logistic regression models will be used to test the difference in the proportion of participants with at least 1 episode of RTI at a 4-month follow-up between the K+ and C groups (primary comparison), adjusted for gender (stratification factor). The effect of the intervention will be estimated between the 2 groups using a model-adjusted odds ratio with a 95% CI. Statistical tests will be 2-sided at a 5% significance level.

Generalized linear regression models will be used on other secondary outcomes to test the group differences at 2- and 4-month follow-ups separately, using a link function appropriate to the distribution of the outcome measure. All regression analyses will be adjusted for baseline outcome value (if available) and gender. The effect of the intervention will be estimated and tested using a model-adjusted point estimate on the group difference with a 95% CI and a 2-sided *P* value. Multiple testing on secondary outcomes will not be adjusted as they are considered exploratory and provide supporting information on the primary outcome. A random-effects mixed model will be used on the outcomes collected repeatedly over time to account for correlated data collected from the same participants and take into account any missing data in maximum likelihood estimates. The consistency of the short- and long-term effects of the dietary intervention will be assessed using the interaction term between the treatment group and visit. The effects of the intervention on predefined subgroups will also be assessed, and separate subgroup analyses will be considered if a sufficient number of participants are recruited.

### Quality Control and Quality Assurance

The SUN study researchers are trained and monitored by an independent academic research organization based in Auckland, New Zealand. The Green Lane Coordinating Centre conducts training and monitoring in accordance with the International Council on Harmonization—Guideline for Good Clinical Practice within the setting of a nutrition intervention trial. The Green Lane Coordinating Centre performs a centralized review of SUN study data (blinded to intervention allocation) and other documents to assess protocol adherence, data accuracy and completeness, source data verification, and review investigator files.

### Data Management and Governance

Each participant is given a unique study identification number, which serves as identification on all forms, questionnaires, and measurements and as a participant identification number on the study database. Only 1 document exists that links participant numbers to personal data. All study data are regarded as confidential and stored electronically using REDCap, hosted by the University of Auckland [20]. All files (electronic and hard copy) will only be available to the key researchers at the University of Auckland performing data collection. After completion of the study, the University of Auckland will retain all data for 10 years after the youngest participant has turned 16 years of age. Access to deidentified data will be available to lead scientists within the wider SUN team for inclusion in analyses.

## Results

Recruitment and data collection did not commence until January 2022 due to the COVID-19 pandemic. Data collection and analyses are expected to conclude in January 2024 and early 2025, respectively.

## Discussion

At 4 months after randomization, our study aims to assess the impact of consuming kūmara (sweet potato) (K) or kūmara with added banana powder containing RS (K+) on the development of the gut microbiome and the prevalence of RTI in comparison to the C. We will also investigate the impact of gut microbiome modifications on immunological development. To our knowledge, this is the first intervention study to examine the effects of a dietary intervention in the CF phase on RTI and the gut microbiome. The study has many advantages over previous designs, including strict inclusion and exclusion criteria, 2 intervention arms and a C (reference group), and a wide range of biological specimen collections.

The completion of a pilot feasibility study prior to the development of the RCT has also enabled the development of robust operational processes that will ensure the successful completion of the trial [16]. The study has been powered to enable the examination of the effect of K and K+ on RTI as this is a tangible clinical outcome; however, the study design also allows us to explore how the intervention impacts the development of the gut microbiome and if the gut microbiome is a mediator between dietary intake and immune development.

There are also several novel exploratory aspects of the study. The first is the collection of breast milk samples, fecal samples, and dietary intake from the infants to examine the role of breast milk and its interaction with food intake on the seeding of the infant’s gut microbiota. The second is the analysis of the infant blood samples to assess the antibody response following oral rotavirus vaccination, which will be complemented by a high-dimensional profiling of the infant’s peripheral immune system; these analyses will enable us to examine the relationship between dietary intake, the gut microbiome, and immune development and function. The third is the collection of objective measurements of several sleep variables important for sleep health [41].

Clinical trials in infants are challenging, and compliance with a dietary intervention and the collection of biological and dietary intake data place a significant burden on caregivers. Previous experience in RCTs in this age group and the completion of the pilot feasibility study provide us with some significant insights into how to support families through the study to ensure retention and completion. There are limited risks to the infants from being involved in the study. The intervention (kūmara) is a common and acceptable complementary food with no known allergenicity. The benefits of using kūmara as a candidate prebiotic food were identified through an in silico reverse genomic approach [42]. This integrated pipeline, based on literature searching, text mining, reverse metabolic analysis, and food database exploration, identified exogenous metabolites required by gut microorganisms potentially capable of enhancing the immune system against infant infections and localized those metabolites in foods and food components.

This study will provide some understanding of the effects of the introduction of a prebiotic complementary food on the development of the gut microbiota, the modulation of immune development, and health in infancy. It will add to the body of growing literature that seeks to expand our understanding of the interactions between diet, gut microbiota, and health in early postnatal life. This knowledge will assist in providing evidence for suitable CF recommendations to maintain an age-appropriate gut microbiome for short- and long-term health.

### Data Management Committee

The principal investigator is responsible for project coordination and oversight of the operational aspects of the trial. A data monitoring committee monitors the adverse events of the study.

## Abbreviations

C: control
CF: complementary feeding
GP: general practitioner
IgA: immunoglobulin A
K: kūmara
K+: kūmara with added banana powder containing resistant starch
REDCap: Research Electronic Data Capture
RS: resistant starch
RTI: respiratory tract infection
SCFA: short-chain fatty acid
SUN: seeding through feeding

## Data Availability

The data sets generated and analyzed during this study are available from the corresponding author on reasonable request through the university Figshare platform [43].
